# The vitamin D_3_ axis in laryngeal cancer: a double-edged sword modulated by estrogen signaling

**DOI:** 10.3389/fimmu.2025.1688589

**Published:** 2025-10-30

**Authors:** Qian Guo, Yicheng Chen, Qiang Zhou, Dong Dong, Shuman Huang, Meirong Shan, Baiquan Zhang, Liuye Pan, Yulin Zhao

**Affiliations:** ^1^ Department of Rhinology, The First Affiliated Hospital of Zhengzhou University, Zhengzhou, China; ^2^ School of Acupuncture-Moxibustion and Tuina, Nanjing University of Chinese Medicine, Nanjing, China; ^3^ Department of Neurosurgery, Shanghai Tenth People’s Hospital of Tongji University, Shanghai, China; ^4^ Department of Geriatrics, First Affiliated Hospital of Zhengzhou University, Zhengzhou, China; ^5^ Department of Respiratory Medicine, The First Affiliated Hospital of Zhengzhou University, Zhengzhou, China; ^6^ Department of Oncology, The Affiliated Hospital of Youjiang Medical University for Nationalities, Baise, China; ^7^ Key Laboratory of Molecular Pathology in Tumors of Guangxi Higher Education Institutions, Affiliated Hospital of Youjiang Medical University for Nationalities, Baise, China

**Keywords:** vitamin D3, laryngeal cancer, cell cycle, estrogen, immunity

## Abstract

Laryngeal cancer remains a formidable clinical challenge, with growing evidence that vitamin D_3_ acts as a potential therapeutic modulator. However, its precise role is complex, largely due to poor understanding of the mechanisms underlying its variable efficacy. This review synthesizes current knowledge to establish a comprehensive framework for vitamin D_3_’s dichotomous role in laryngeal carcinogenesis. First, we clarify its two distinct mechanisms of action: (i) directly inhibiting laryngeal cancer cell proliferation and survival via the canonical vitamin D receptor (VDR) axis—triggering G_0_/G_1_ cell cycle arrest, inducing apoptosis, and reversing epithelial-mesenchymal transition (EMT); (ii) indirectly exerting anti-tumor effects by reprogramming the tumor immune microenvironment, including enhancing cytotoxicity of CD8^+^ T and natural killer (NK) cells, promoting dendritic cell maturation, and suppressing key inflammatory pathways such as the COX-2/PGE_2_ axis. Subsequently, we propose that the net effect of vitamin D_3_ signaling is context-dependent and double-edged, determined mainly by host-intrinsic and viral factors—most notably estrogen receptor α (ERα66) expression. Specifically, vitamin D_3_-related products promote cell growth in ERα66-positive laryngeal cancer cell lines, but suppress growth in ERα66-negative lines, thereby aiding cancer therapy. This integration provides a nuanced paradigm, highlighting the need for biomarker-driven patient stratification to harness vitamin D_3_’s therapeutic potential in laryngeal cancer.

## 1 Introduction

Head and neck malignancies rank seventh among the most prevalent cancers worldwide, with laryngeal carcinoma accounting for approximately one-fifth of these cases (1). Laryngeal cancer is a malignant neoplasm originating from laryngeal tissues and constitutes a major global health burden (2). According to 2022 data released by the World Health Organization, there were an estimated 188960 new cases of laryngeal cancer globally, resulting in approximately 103216 deaths. Both incidence and mortality display pronounced sex disparities. Epidemiological studies indicate a male-to-female incidence ratio of roughly 4:1. In certain regions, the incidence among men is markedly higher, with population-based surveys reporting ratios approaching 10:1 (3). A similar trend is observed in mortality, with more men than women succumbing to laryngeal cancer, likely attributable to differences in lifestyle factors and biological characteristics between sexes (3). Laryngeal cancer can be classified according to anatomical location and histological features. Anatomically, it is divided into three main types: supraglottic carcinoma, glottic carcinoma, and subglottic carcinoma (4). Histologically, most laryngeal cancers are identified as squamous cell carcinomas originating from the laryngeal squamous epithelium (5). Less common histological variants include adenocarcinomas (originating from glandular cells) and sarcomas (originating in connective tissues, including muscle and cartilage) (5). Vitamin D encompasses several fat-soluble compounds that are essential micronutrients required for maintaining human health. It comprises vitamin D_2_ and vitamin D_3_; the former is predominantly sourced from plants following ultraviolet activation, while the latter is chiefly acquired from animal products or produced endogenously in the skin in response to ultraviolet radiation. The present article centers on the function of vitamin D_3_ (6). Vitamin D_3_ itself is inactive and requires sequential hydroxylation to generate active metabolites. Specifically, upon entering the circulation, vitamin D_3_ associates with vitamin D-binding protein (DBP), facilitating its transport to the liver (7). Within the liver, vitamin D_3_ undergoes hydroxylation by 25-hydroxylase (encoded by CYP2R1), resulting in the formation of 25-hydroxyvitamin D_3_, the main storage form. Following hepatic conversion, the 25-(OH)D_3_-DBP complex is transported to renal tissue, where CYP27B1-encoded 1α-hydroxylase catalyzes the final hydroxylation step, producing the biologically active hormone 1,25-dihydroxyvitamin D_3_. While another enzyme in the kidney, 24-hydroxylase (encoded by the gene CYP24A1), can hydroxylate it into 24R,25-(OH)_2_D_3_(an active native conformer of 24,25-(OH)_2_D_3_). After exerting its biological effects in cells and tissues, 1,25-(OH)_2_D_3_ is further hydroxylated by 24-hydroxylase (encoded by CYP24A1) into inactive 1,24,25-trihydroxyvitamin D_3_ (1,24,25-(OH)_3_D_3_) in liver prior to excretion; this constitutes the classical pathway of vitamin D_3_ metabolism (8–10). However, accumulating evidence indicates that vitamin D_3_ can also be synthesized locally via paracrine pathways. Dendritic cells and macrophages secrete 1,25-(OH)_2_D_3_ to suppress excessive immunity or modulate cell differentiation (11). In the cutaneous microenvironment, keratinocytes together with skin-resident immune populations locally synthesize 1,25-dihydroxyvitamin D_3_, thereby orchestrating epidermal turnover, lineage-specific differentiation, inflammatory tone, and tissue repair after injury (12). Tissues including breast, prostate, pancreas, and larynx possess local vitamin D_3_-converting capacity, potentially participating in cell proliferation control and tissue homeostasis (13–17). Moreover, whereas 24R,25-(OH)_2_D_3_ was traditionally considered metabolically inert (18), recent studies reveal its biological activity in laryngeal cancer cells, modulating cell-cycle progression (19). Clinical evidence demonstrates that reduced vitamin D_3_ levels correlate significantly with poor survival in advanced laryngeal cancer patients undergoing total laryngectomy (20, 21), suggesting an important role of vitamin D_3_ in laryngeal cancer pathogenesis and therapy. “Vitamin D_3_ axis” in the title refers to the entire pathway, encompassing the dietary intake and endogenous synthesis of vitamin D_3_, its metabolic activation into 25-hydroxyvitamin D_3_ and the hormonal form 1,25-dihydroxyvitamin D_3_, VDR, and the subsequent downstream genomic and non-genomic signaling events. As an essential fat-soluble vitamin, vitamin D_3_ participates in laryngeal cancer pathophysiology via two primary mechanisms: First, direct regulation of tumor cell biology, and second, modulation of the host immune microenvironment (22). For the former, we focus on cell-cycle control and other cancer-cell-intrinsic mechanisms; for the latter, on immune-cell modulation and key immunoregulatory molecules. We also highlight estrogenic influences, as the hormone-sensitivity status of laryngeal cancer remains unresolved. Additionally, whether laryngeal cancer is hormone-sensitive remains debated. Although traditional views attribute sex disparities in incidence mainly to differential smoking rates, emerging evidence implicates estrogen signaling (23). Notably, when laryngeal cancer cells express estrogen receptors, estrogen may interfere with vitamin D_3_ bioactivity and modulate the paracrine processes of vitamin D_3_-active metabolites within the tumor microenvironment (24, 25). This underexplored crosstalk is clinically significant for understanding sex-based differences in laryngeal cancer and for developing sex-specific therapeutic strategies.

## 2 Vitamin D_3_ regulates laryngeal cancer cell growth and migration

### 2.1 Vitamin D_3_ influences the laryngeal cancer cell cycle

The biologically active form of D_3_(1,25-(OH)_2_D_3_)binds to the VDR and arrests cells in the G_0_/G_1_ phase, thereby inhibiting proliferation and inducing differentiation in various malignancies, such as cell lines of head and neck squamous cell carcinoma (SCCHN) (26). Specifically, it markedly induces expression of the cell-cycle inhibitors p21 and p27; p21 and p27 bind and inhibit CDK2–cyclin E and CDK2–cyclin A complexes, preventing phosphorylation of retinoblastoma protein (Rb). Hypophosphorylated Rb sequesters the transcription factor E2F, suppressing expression of S-phase genes (e.g., those required for DNA replication) and blocking G_1_-to-S transition, thus arresting cells in G_0_/G_1_ (27–30). 1,25-(OH)_2_D_3_ also promotes p38 phosphorylation to its active form; activated p38 indirectly controls the cell cycle by inducing p21 expression (31). Furthermore, the vitamin D_3_ analogue EB1089 up-regulates p57 expression and synergizes with p21 and other cell-cycle inhibitors to induce G_1_ arrest and inhibit cancer cell proliferation (32). Vitamin D_3_ arrests the laryngeal cancer cell cycle as a cornerstone of its antiproliferative effect, yet concurrently targets multiple signaling axes and phenotypic plasticity to orchestrate a multi-dimensional suppression of tumor cell behavior.

### 2.2 Additional regulatory mechanisms

Treatment with 1,25-(OH)_2_D_3_ exerts a significant inhibitory effect on the IL-6–JAK–STAT3 signaling pathway in cancer cells (33). Although direct studies in laryngeal cancer are lacking, existing evidence suggests that: VDR is expressed in laryngeal cancer cells and influences tumorigenesis and prognosis (21). VDR protein binds the Jak2 promoter, transcriptionally down-regulating Jak2 expression (34). When 1,25-(OH)_2_D_3_ binds, the VDR–RXR heterodimer can competitively bind the dimerization domain of STAT3, preventing formation of functional p-STAT3 dimers. The VDR–RXR dimer can also occupy NF-κB binding sites within the IL-6 promoter to inhibit NF-κB-mediated transcriptional activation of IL-6 (35). At appropriate concentrations, 1,25-(OH)_2_D_3_ time- and dose-dependently inhibits the PI3K/AKT/Bcl-2 pathway, inducing apoptosis in Hep-2 laryngeal carcinoma cells (36). Epithelial–mesenchymal transition (EMT) denotes a dynamic process whereby epithelial cells lose epithelial characteristics and acquire mesenchymal phenotypes, thereby enhancing migratory and invasive capacities. *In vitro* knockdown of Snail inhibits EMT in LSCC cells via the VDR signaling pathway (37, 38). Snail directly binds three E-boxes within the promoter of the epithelial marker E-cadherin, repressing its expression while up-regulating mesenchymal markers such as matrix metalloproteinases (MMP)-2 and MMP-9, disrupting epithelial cell–cell contacts and conferring increased motility, thereby facilitating invasion and metastasis (37, 39). As the tumor microenvironment concept matures, peritumoral cells gain prominence, and vitamin D_3_’s modulation of these bystanders in laryngeal cancer is equally pivotal.

## 3 Vitamin D_3_ modulates the laryngeal cancer immune microenvironment

### 3.1 Vitamin D_3_ enhances immune cell infiltration and differentiation

**Figure 1:**
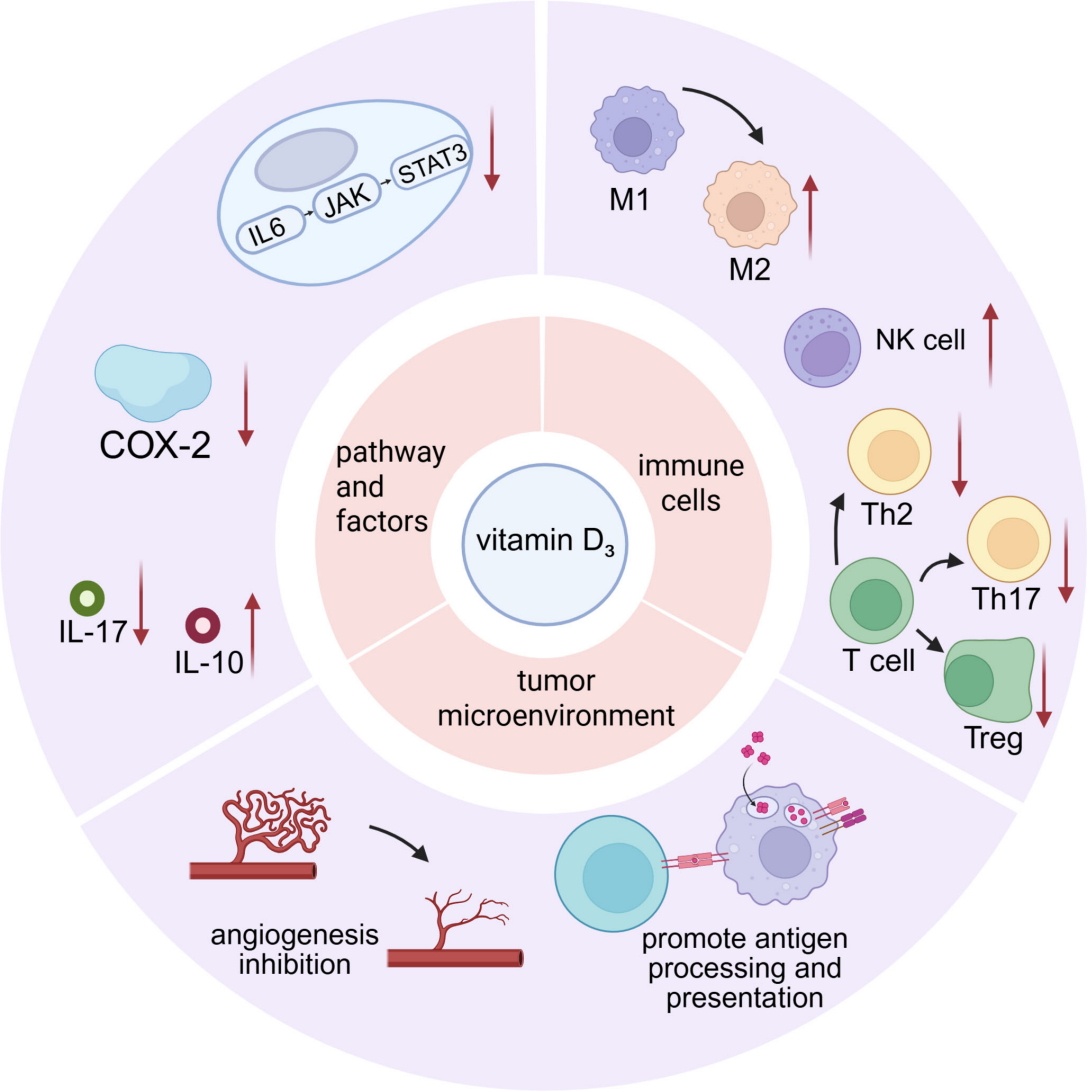
The multifaceted effects of vitamin D_3_ on the cells, molecules, and immune microenvironment.

Vitamin D_3_ promotes infiltration of CD3^+^, CD8^+^, and NKR-P1C^+^ immune cells within the tumor microenvironment, reduces M2 macrophages and regulatory T cells (Tregs), and thus impedes tumor immune escape (33, 40). An elevated count of immunosuppressive CD34^+^ progenitor cells is detected in both peripheral blood and tumor tissues. Tumor-derived granulocyte-macrophage colony-stimulating factor (GM-CSF) induces expansion of these CD34^+^ cells, and tumor-secreted vascular endothelial growth factor (VEGF) chemoattracts them to the tumor site (41). These CD34^+^ cells suppress autologous T-cell function; removal of CD34^+^ cells markedly enhances IFN-γ production by T cells stimulated with anti-CD3 antibody and low-dose IL-2 (41, 42). Treatment with 1,25-(OH)_2_D_3_ decreases intratumoral CD34^+^ progenitor cells in HNSCC patients, promotes their differentiation into dendritic cells, and increases intratumoral T-cell infiltration (43, 44), supporting further investigation of 1,25-(OH)_2_D_3_-mediated immunomodulation within the tumor microenvironment (42, 44, 45). 25-(OH)D_3_ elevates HLA-DR expression and increases plasma IL-12 and IFN-γ levels while improving T-cell proliferative responses. 1,25-(OH)_2_D_3_ induces expression of the pattern-recognition receptor CD14 gene in epithelial cells (46) and drives the monocytic cell line HL60 toward monocyte or macrophage differentiation. The T1/ST2 protein (IL-1 receptor family member) gene is also strongly induced; murine knockout studies demonstrate that T1/ST2 signaling is essential for Th2 differentiation (46). 1,25-(OH)_2_D_3_ increases CD4^+^ and CD8^+^ T-cell levels and augments intratumoral populations expressing the early activation marker CD69. Additionally, 1,25-(OH)_2_D_3_ reduces tumor angiogenesis, thereby inhibiting tumor progression and metastasis (**Figure 1**). Next, we dissect at the molecular level how vitamin D_3_ precisely reprograms immune microenvironmental cells: (i) by modulating cyclo-oxygenase-2 activity and (ii) by reshaping the expression and secretion of key inflammatory mediators.

### 3.2 COX-2 plays a pivotal role in the immune response modulated by vitamin D_3_

Cyclo-oxygenase-2 (COX-2) is an enzyme closely linked to immune responses. Its catalytic product PGE_2_ drives a shift of helper T cells from Th1 to Th2; imbalance of Th1/Th2 ratios causes immune dysregulation. PGE_2_ also polarizes macrophages from M1 to M2 phenotype; M2 macrophages possess immunosuppressive properties, secreting IL-10 and TGF-β to inhibit antitumor immunity. Furthermore, PGE_2_ induces development of Tregs, Th17 cells, and myeloid-derived suppressor cells (MDSCs) while suppressing dendritic and NK cell functions, thereby fostering a tumor-permissive immune milieu (40, 47, 48). 1,25-(OH)_2_D_3_ alone down-regulates overexpressed COX-2 in both tumor and immune cells, reducing production of inflammatory mediators such as prostaglandin E_2_ (PGE_2_) and thereby alleviating immunosuppression and inflammation (1). However, when combined with the commonly used chemotherapeutic agent cisplatin, vitamin D_3_ up-regulates COX-2 expression within the laryngeal mucosal epithelial stroma, potentially exacerbating mucosal injury and inflammation (49) (**Figure 1**).

### 3.3 Vitamin D_3_ modulates tumor microenvironment via pro-inflammatory cytokines

While delineating vitamin D_3_ mechanisms, we interrogate the determinants of its efficacy. Beyond smoking and HPV, we focus on sex steroids—an emerging but understudied modulator of laryngeal cancer outcome. As stated previously, IL-6 activates the IL-6–JAK–STAT3 pathway to promote tumor growth. 1,25-(OH)_2_D_3_ downregulates pro-inflammatory cytokines (IL-6, IL-17, TNF-α) and the immunosuppressive cytokine IL-1, thereby mitigating cancer-associated chronic inflammation. As an immunosuppressive cytokine, IL-10 limits T-cell activation via suppression of dendritic cell maturation and antigen presentation (50, 51). 1,25-(OH)_2_D_3_ increases HLA-DR expression, elevates plasma IL-12 and IFN-γ levels, and enhances T-cell proliferation (45). Within the tumor milieu, Th17 (CD4^+^) cells exhibit pro-inflammatory and pro-angiogenic properties and can differentiate into immunosuppressive Tregs (52). Th17 cells exert their effects primarily via IL-17 production, and 1,25-(OH)_2_D_3_ reduces IL-17 levels, thereby attenuating Th17-mediated disease progression (51). Additionally, studies reveal discordant cytokine responses to 1,25-(OH)_2_D_3_ between tumor tissue and peripheral blood; plasma cytokine profiles may not accurately reflect intratumoral immune status (53) (**Figure 1**).

## 4 Estrogen is a critical regulator of vitamin D_3_ actions on tumor and immune cells

**Figure 2:**
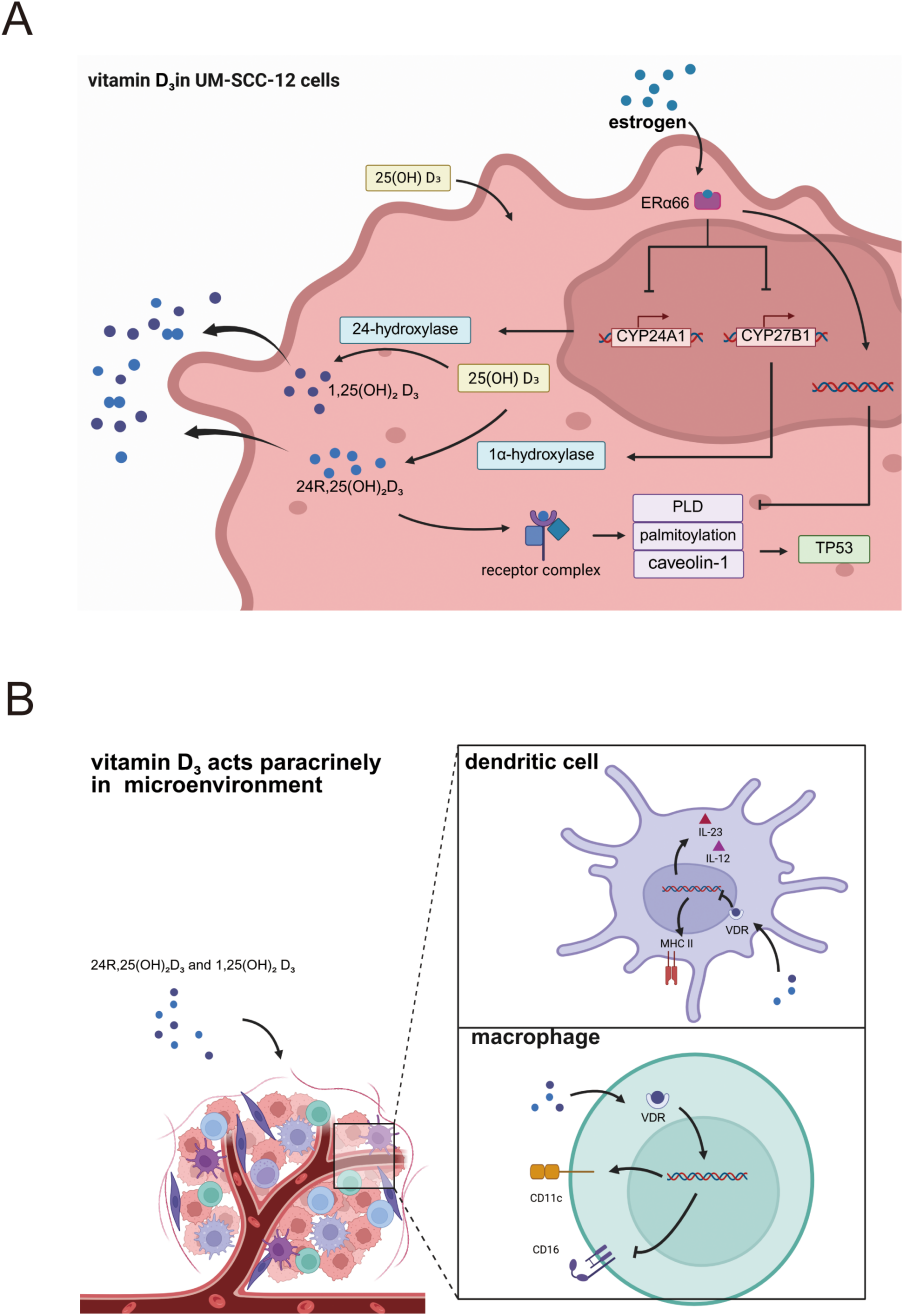
**(A)** Mechanisms of vitamin D_3_ action in laryngeal cancer cells expressing estrogen receptors. **(B)** Paracrine vitamin D_3_ shapes immune cells within the laryngeal-cancer immune microenvironment.

The vitamin D_3_ derivative 24R,25-dihydroxyvitamin D_3_ exerts cell-type-specific effects on laryngeal carcinoma cells, which are modulated by the status of the estrogen receptor α66 (ERα66). 24R,25-dihydroxyvitamin D_3_, when acting on human head and neck squamous cell carcinoma cell line with estrogen receptor α66 negativity (UM-SCC-11A cells), suppresses proliferation, upregulates apoptosis-related markers (TUNEL positivity, p53 expression, and BAX/BCL2 ratio), and downregulates metastasis-associated markers, with these effects collectively reflecting its tumor-suppressive capacity (17). Conversely, in human head and neck squamous cell carcinoma cell line with estrogen receptor α66 positivity (UM-SCC-12 cells), 24R,25-(OH)_2_D_3_ promotes multiplication, reduces DNA fragmentation (TUNEL-negative), and increases total p53, reflecting tumor promotion (19). Previous research has established that laryngeal cancer cells exhibit individual differences in their responsiveness to vitamin D_3_ supplementation, with recent investigations identifying ERα66 expression as a key determinant of this variability. Laryngeal cancer cells possess the capacity for local vitamin D_3_ metabolism, a process that in turn modulates cellular fate and shapes the tumor microenvironment. Estrogen modulates expression of vitamin D_3_ hydroxylases (25), and laryngeal cancer cells possess the capacity to synthesize estrogen, exerting autocrine and paracrine effects (23). Hydroxylases CYP27B1 and CYP24A1 are critical enzymes in vitamin D_3_ metabolism: CYP27B1 converts 25-(OH)D_3_ to 1,25-(OH)_2_D_3_, whereas CYP24A1 hydroxylates 25-(OH)D_3_ to 24R,25-(OH)_2_D_3_ and further converts 1,25-(OH)_2_D_3_ to 1,24,25-(OH)_3_D_3_ (24). Both enzymes are expressed in laryngeal cancer cells; ERα66 exerts an inhibitory effect on these activities, resulting in decreased biosynthesis of active 1,25-(OH)_2_D_3_ and 24R,25-(OH)_2_D_3_ in UM-SCC-12 cells relative to UM-SCC-11A cells; this, in turn, impacts tumor progression and immune cell functionality within the microenvironment. Investigations have demonstrated that 24R,25-(OH)_2_D_3_ exerts its effects through the phospholipase D (PLD), caveolae, and palmitoylation pathways. For example, 24R,25-(OH)_2_D_3_ increases PLD activity in UM-SCC-12 cells but decreases it in UM-SCC-11A cells; inhibiting PLD activity or palmitoylation, or silencing caveolin-1 expression, alters p53 levels. p53 is a key cell-cycle checkpoint molecule, and these perturbations modulate tumor behavior—for instance, promoting p21 expression and G1/G2 arrest in UM-SCC-11A cells (54). In UM-SCC-12 cells, 24R,25-(OH)_2_D_3_ docks with a membrane complex composed of TLCD3B2, VDR and protein disulfide-isomerase A3 (PDIA3); this interaction is palmitoylation-dependent and requires coordinated PLD–PI3K–LPAR activity. In contrast, UM-SCC-11A cells utilize a VDR–PDIA3–ROR2 complex that triggers endosomal signaling cascades, the molecular details of which remain undefined (55). Additionally, estrogen via ERα66 modulates paracrine effects of vitamin D_3_ and its metabolites, influencing immune cell infiltration and differentiation within the tumor microenvironment. The paracrine effects of vitamin D_3_ and its active metabolites on cells modulate immune cell infiltration and differentiation within the tumor microenvironment. Specifically, active vitamin D_3_ secreted into the tumor milieu downregulates MHC class II molecules on dendritic cells (DCs), thereby attenuating their antigen-presenting capacity. Additionally, active vitamin D_3_ or its analogs suppress DC-derived cytokine production, particularly interleukin (IL)-12—which directs helper T-cell differentiation toward the Th1 phenotype—and IL-23, which promotes Th17 differentiation (56). In macrophages, active vitamin D_3_ primarily regulates polarization, shifting macrophages from the M2 to the M1 phenotype, as evidenced by upregulated M1 markers CD11c and concomitant suppression of M2 markers CD16 (40) (**Figure 2**).

Research directly investigating the role of 24R,25-(OH)_2_D_3_ in laryngeal cancer is limited. Therefore, its potential molecular mechanisms in laryngeal cancer cells and their associated immune cells are largely extrapolated from studies in other cell types, such as osteoblasts. The proposed mechanism via the canonical VDR-dependent pathway is as follows: Upon cellular entry, 24R,25-(OH)_2_D_3_ first binds to the Vitamin D Receptor (VDR), forming a 24R,25-(OH)_2_D_3_-VDR complex (57). Subsequently, this complex must assemble with the Retinoid X Receptor (RXR) to form a heterodimer, a critical structural step for initiating downstream transcriptional regulation (57). The 24R,25-(OH)_2_D_3_-VDR-RXR heterodimer then targets and binds to Vitamin D Response Elements (VDREs) located in the promoter regions of target genes (58). However, due to its relatively weak binding affinity, this process often requires the assistance of Nuclear Auxiliary Factors (NAFs) to enhance binding efficiency. Ultimately, the heterodimer, once bound to the VDRE, modulates the transcriptional activity of target genes by recruiting co-activator or co-repressor complexes. This, in turn, influences the expression of downstream genes, thereby regulating biological functions such as cell proliferation, differentiation, and immune modulation (59). The mechanisms of action for 24R,25-(OH)_2_D_3_ also encompass a VDR-independent pathway. For instance, 24R,25-(OH)_2_D_3_ can bind to the cell membrane of chondrocytes, leading to the activation of Protein Kinase C (PKC). This subsequently influences the Mitogen-Activated Protein Kinase (MAPK) pathway, ultimately resulting in new gene expression through a process independent of VDR (60). Furthermore, the specific molecular interplay between VDR and the Estrogen Receptor (ER) is a key area of investigation. Insights can be drawn from breast cancer, another hormone-dependent malignancy analogous to laryngeal cancer. In breast cancer cells, Estrogen-Related Receptor alpha (ERRα) regulates gene expression and transcription through two primary mechanisms. First, ERRα can directly bind to the promoters of the CYP24A1, ERα, and aromatase (CYP19A1) genes, or recruit co-activators like p300 to alter chromatin conformation (61, 62). These actions respectively promote: the degradation of active vitamin D by CYP24A1, thereby interfering with calcitriol-VDR transcription, the enhancement of estrogen signaling by ERα, and the elevation of local estrogen levels by aromatase—all of which favor cancer cell growth. Second, the Ligand-Binding Domain (LBD) of ERRα binds to the LxxLL/LLxxL motifs of PGC-1α (63, 64). This complex then recruits the CBC and Mediator complexes via the CBM and RS domains of PGC-1α. Subsequently, it assembles with VDR to form a larger transcriptional complex. Upon binding to the target gene’s VDRE, this complex efficiently promotes target gene expression by recruiting RNA Polymerase II, facilitating transcriptional elongation, and preventing premature termination, thereby influencing cancer cell behavior (65). However, it is crucial to note that these molecular mechanisms cannot be directly extrapolated from breast cancer to laryngeal cancer, as the two malignancies exhibit distinct and sometimes contradictory experimental and clinical manifestations.

## 5 Discussion

Vitamin D_3_ orchestrates a complex regulatory network in laryngeal cancer initiation, progression, and immune microenvironmental remodeling. This study systematically delineates its dual-pathway impact: (i) via the canonical vitamin D receptor axis, directly modulating tumor cell biology, including p21/p27/p57-dependent G_0_/G_1_ arrest (27–30, 32), PI3K/AKT/Bcl-2 inhibition-mediated apoptosis (36), and Snail down-regulation-reversed EMT (37, 39); and (ii) by reshaping the immune microenvironment, it exerts anti-tumor effects through reducing CD34^+^ immunosuppressive progenitor infiltration and promoting their differentiation into dendritic cells (42–44), enhancing CD8^+^ T-cell and NK-cell activity (33, 45), and suppressing the COX-2/PGE_2_ pathway and pro-inflammatory cytokines IL-6/IL-17/TNF-α (33, 47, 51). Notably, these regulatory effects are constrained by a triad of factors, namely ERα66 status, HPV infection, and VDR/CYP24A1 polymorphisms (17, 19, 21, 33), constituting the molecular basis for heterogeneous therapeutic responsiveness. ERα66, a key mediator of sexual dimorphism in laryngeal cancer, plays a critical role in regulating vitamin D_3_ metabolism and its biological functions. Specifically, in ERα66-negative cells (UM-SCC-11A), 24R,25-(OH)_2_D_3_ inhibits cellular proliferation and triggers apoptosis through the activation of the p53/p21 pathway (17, 19), whereas in ERα66-positive cells (UM-SCC-12), the same metabolite promotes tumor progression (19). This paradoxical effect arises from ERα66-mediated suppression of local vitamin D_3_ hydroxylases: ERα66 downregulates CYP27B1 and CYP24A1 activities (23–25), diminishing the generation of anti-tumoral 1,25-(OH)_2_D_3_ and disrupting paracrine control of immune cells by vitamin D_3_ metabolites (54). These findings offer a mechanistic explanation for the higher incidence of laryngeal cancer in males and underscore the centrality of estrogen–VDR crosstalk in microenvironmental remodeling (3, 23). However, direct evidence demonstrating that estrogen modulates vitamin D_3_ signaling in laryngeal cancer remains scarce. Most inferences are extrapolated from breast-cancer models. More nuanced and context-specific investigations are therefore urgently required. Furthermore, smoking, body weight, gender, host genetic polymorphisms, and HPV status are also recognized as crucial factors influencing the progression and prognosis of laryngeal cancer (66). A significant proportion of patients with this malignancy have a history of smoking (67). Moreover, compared with current smokers, former smokers exhibit a substantially reduced laryngeal-cancer risk (68). Studies have indicated that men with lower abdominal adiposity are at a greater risk of developing laryngeal cancer than females with higher abdominal adiposity (69). HPV status also dictates therapeutic responsiveness: 1,25-(OH)_2_D_3_ suppresses the MYC oncogenic program in HPV-positive cells but may activate it in HPV-negative contexts 1 (33). Associations between VDR/CYP24A1 polymorphisms and the recurrence risk of glottic carcinoma further emphasize the need for genotype-guided therapy (21). Prospective studies further indicate that the therapeutic and prognostic impact of vitamin D_3_ in laryngeal cancer exhibits substantial inter-individual heterogeneity (70). These factors should serve as stratification criteria. Based on the foregoing evidence, the following clinically actionable strategies for laryngeal cancer can be advanced: patients should be pre-stratified based on ERα66 expression detected via immunohistochemistry: for ERα66-negative patients, 24R,25-(OH)_2_D_3_ should be administered as adjuvant therapy; for ERα66-positive patients, vitamin D_3_ analogs (e.g., EB1089) should be combined with ERα66 inhibitors. The core goal of these treatment strategies is to reverse the suppression of CYP27B1/CYP24A1 and thereby restore the biosynthesis of active metabolites (71). It is possible to combine vitamin D_3_ with immune checkpoint inhibitors (ICIs): in resectable cases, use high-dose 25-(OH)D_3_ for 2–4 weeks pre-ICI to enhance CD8^+^ T-cell infiltration and suppress COX-2/PGE_2_; for unresectable patients, further stratify by HPV (prioritizing HPV-positive cohorts, where 1,25-(OH)_2_D_3_ suppresses MYC) to optimize benefit (72, 73). In the clinical setting, CYP27B1/CYP24A1-targeted agents may also be considere. Test CYP24A1 inhibitors in patients with VDR/CYP24A1 polymorphisms, monitoring intratumoral 1,25-(OH)_2_D_3_ levels and Ki-67 to validate “metabolite-guided” dosing (74). Clinical translation of vitamin D_3_ faces multiple contradictions. Although low vitamin D_3_ levels correlate with poor prognosis (20, 21), several limitations exist. First, combined use with cisplatin may exacerbate mucositis via COX-2 upregulation (49), necessitating cautious combination strategies. Second, peripheral vitamin D_3_ levels and cytokine profiles poorly mirror the intratumoral immune landscape (53), limiting the utility of systemic biomarkers. Finally, head and neck cancer patients frequently exhibit vitamin D_3_ deficiency, yet supplementation strategies must be tailored to ERα66 strata. To date, multiple clinical studies have confirmed that vitamin D_3_ can improve the prognostic survival rate of patients with laryngeal cancer, while vitamin D_3_ deficiency is a risk factor for laryngeal cancer development (75, 76). More targeted clinical studies are needed to further enrich the evidence base in this field.

## 6 Conclusion

Vitamin D_3_ exerts a profound yet dichotomous influence on laryngeal cancer, acting as a master regulator at the nexus of direct tumor cell biology and immune microenvironment remodeling. Its function transcends a simple anti-proliferative role; instead, it operates as a context-dependent ‘rheostat,’ where its ultimate anti-tumor efficacy is contingent upon the tumor’s specific molecular landscape, notably the host’s ERα66 expression. This understanding necessitates a paradigm shift from a ‘one-size-fits-all’ supplementation strategy towards precision-guided interventions. Future research must prioritize clinical trials stratified by these biomarkers to validate therapeutic efficacy. Furthermore, exploring synergistic combinations of vitamin D_3_ with immune checkpoint inhibitors, and developing novel agents targeting key metabolic enzymes like CYP27B1/CYP24A1 to optimize local active metabolite concentrations, represent promising avenues. Integrating these multi-level insights will be pivotal for translating the complex biology of vitamin D_3_ into tangible, personalized therapeutic benefits for patients with laryngeal cancer.
